# Comparative diagnostic accuracy of artificial intelligence-derived risk stratification versus conventional risk stratification methods in pulmonary hypertension patients: a systematic review and meta-analysis

**DOI:** 10.3389/frai.2025.1692829

**Published:** 2025-11-19

**Authors:** Faizan Ahmed, Faseeh Haider, Muhammad Arham, Allah Dad, Kinza Bakht, Muhammad Moseeb Ali Hashim, Paweł Łajczak, Muhammad Hassan, Fatima Binte Athar, Muhammad Adnan, Muhammad Usman, Najam Gohar, Tehmasp Mirza, Mushood Ahmed, Mark Moshiyakhov, Brett Sealove, Swapnil Patel, Jesus Almendral, Mohamed Bakr, Yasar Sattar, Fawaz Alenezi

**Affiliations:** 1 Department of Medicine, Hackensack Meridian Health, Jersey Shore University Medical Center, Neptune, NJ, United States; 2 Department of Medicine, Allama Iqbal Medical College, Lahore, Pakistan; 3 Sheikh Zayed Medical College, Rahim Yar Khan, Pakistan; 4 University of Missouri, Columbia, MO, United States; 5 Medical University of Silesia, Katowice, Poland; 6 Karachi Medical and Dental College, Karachi, Pakistan; 7 Mission Hospital, Asheville, NC, United States; 8 Ameer-ud-Din Medical College, Lahore, Pakistan; 9 Shalamar Medical and Dental College, Lahore, Pakistan; 10 Rawalpindi Medical University, Rawalpindi, Pakistan; 11 Department of Interventional Cardiology, Tidal Health, Salisbury, MD, United States; 12 Division of Cardiology, Department of Medicine, Duke University School of Medicine, Durham, NC, United States

**Keywords:** AI—artificial intelligence, deep learning, diagnostic accuracy, AI prediction, risk strategies, pulmonary hypertension

## Abstract

**Background:**

Accurate risk stratification in pulmonary hypertension (PH) is integral for optimizing therapeutic strategies and improving patient outcomes. Recent artificial intelligence (AI) models have demonstrated notable efficacy in risk stratification of PH, achieving area under the curve (AUC) values of 0.94 and 0.81 in internal and external validation cohorts, respectively. This meta-analysis aims to demonstrate the effectiveness of AI models in the risk stratification of PH by comparing their performance to conventional risk stratification methods.

**Methods:**

A systematic search of five databases (PubMed, Embase, ScienceDirect, Scopus, and the Cochrane Library) was conducted from inception to March 2025. Statistical analysis was performed in R (version 2024.12.1 + 563) using 2 × 2 contingency data. Sensitivity, specificity, and diagnostic odds ratio (DOR) were pooled using a bivariate random-effects model (reitsma from the mada package), while the AUC was meta-analyzed using logit-transformed values via the metagen() function from the meta package.

**Results:**

Six studies were included in the final synthesis, comprising 14,095 patients: 4,481 in internal test datasets and 4,948 in external datasets. AI risk stratification models showed significant performance with a logit mean difference of 0.26 (95% CI 0.09–0.43; *p* = 0.31), having low heterogeneity (*I*^2^ = 14.3%) as compared to conventional methods. Furthermore, pooled sensitivity and specificity were 0.77 (95% CI 0.74–0.79) and 0.72 (95% CI 0.70–0.75) in favor of AI methods, respectively. The heterogeneities for pooled sensitivity and specificity were 57.1% (*p* = 0.04) and 91.8% (*p* < 0.0001), underscoring high variability across all studies. Finally, DOR was substantially high, 8.53 (6.59–11.04) in favor of AI models with a high heterogeneity of 73.6% (*p* = 0.002). Heterogeneity (I2) for pooled sensitivity went to 25.9% after excluding a major outlier, but it remained high for pooled specificity and DOR upon leave-one-out sensitivity analysis.

**Conclusion:**

Artificial intelligence-based risk stratification demonstrates significantly higher diagnostic performance compared to conventional methods in pulmonary hypertension. The higher pooled AUC, sensitivity, specificity, and DOR highlight AI’s potential to enhance predictive accuracy, guiding better treatment strategies. Nonetheless, more superior quality studies are needed to validate AI models for clinical integration.

## Introduction

Pulmonary hypertension (PH) is a progressive, life-threatening condition characterized by elevated pulmonary vascular resistance, which can lead to right ventricular dysfunction. Accurate risk stratification is essential in PH, as it guides therapeutic decisions, monitors disease progression, and predicts outcomes such as mortality and hospitalization (Oliveros et al., 2025). Risk assessment can be performed using various measures such as the 6-min walk test, cardiopulmonary exercise testing, NT-pro BNP, and multiple chest imaging variables (Ahmed et al., 2023; Benza et al., 2021). Traditional risk scores—such as the REVEAL (Registry to Evaluate Early and Long-term Pulmonary Arterial Hypertension Disease Management), European Society of Cardiology/European Respiratory Society Risk Stratification Model (ESC/ERS), and COMPERA (Comparative, Prospective European Registry of Adult Patients with Pulmonary Arterial Hypertension) models—have long served as clinical tools for these purposes (Pausch et al., 2023; McLaughlin, 2024). However, these models often rely on linear assumptions and predefined variables, which may limit their predictive accuracy in the context of complex, heterogeneous patient populations (Pausch et al., 2023).

Recent advances in artificial intelligence (AI) and machine learning (ML) have opened new avenues for precision medicine in cardiopulmonary diseases, including PH. AI-based models have demonstrated the potential to process high-dimensional data, identify hidden patterns, and generate individualized risk predictions with greater accuracy than conventional scoring systems (Attaripour Esfahani, 2025; Siontis, 2022). Previous systematic reviews comparing AI and conventional risk models in pulmonary hypertension or related cardiopulmonary disorders have reported encouraging results; however, many lacked consistent validation, comprehensive external testing, or pooled diagnostic estimates (Attaripour Esfahani, 2025; Imai, 2024; Park, 2023; Siontis, 2022).

Despite promising results, the comparative performance of AI-based models versus traditional risk stratification tools remains uncertain due to variability in study designs, data sources, outcome measures, and validation strategies. Therefore, we conducted a systematic review and meta-analysis to evaluate the prognostic performance of AI models relative to conventional scoring systems in predicting clinical outcomes among patients with PH.

## Materials and methods

### Protocol

This meta-analysis followed the guidelines set by reporting recommendations of the Preferred Reporting Items for Systematic Reviews and Meta-Analyses for Diagnostic Test Accuracy Studies Statement (PRISMA-DTA) (McInnes et al., 2018).

### Data sources and search strategy

We conducted a comprehensive literature search across five databases: PubMed, Embase, ScienceDirect, Scopus, and the Cochrane Library, which yielded 503 results in the initial search. After removing 44 duplicates, 459 records remained for title/abstract screening. Nineteen studies were sought for full-text assessment, of which six studies met the inclusion criteria and were included in the review. The search strategy included all the keywords and medical subject headings (MeSH) terms in combination with the Boolean operators “AND” and “OR” given in Supplementary Table S1. Each study was independently screened for final inclusion by AD and KB. Any disagreements between the two reviewers were resolved through discussion with a third neutral reviewer, FH. We also searched for references to the retrieved articles and indexed abstracts to include any relevant studies that may have been missed during the search. After this, six studies that fit our inclusion criteria were finalized.

### Inclusion criteria

We included studies enrolling patients with pulmonary hypertension (PH) who underwent risk stratification using clinical and functional variables (6-min walk distance, 6MWD; cardiopulmonary exercise testing, CPET), biomarkers (N-terminal pro-B-type natriuretic peptide, NT-proBNP; troponin-T), echocardiography (tricuspid annular plane systolic excursion, TAPSE; systolic pulmonary artery pressure, sPAP), invasive hemodynamics (mean pulmonary arterial pressure, mPAP; cardiac output, CO; pulmonary vascular resistance, PVR; right atrial pressure, RAP), cardiac magnetic resonance imaging (CMR; right ventricular ejection fraction, RVEF; stroke volume index, SVI), other imaging modalities as reported, and established risk scores (REVEAL 2.0; French Pulmonary Hypertension Registry, FPHR; COMPERA; Swedish Pulmonary Arterial Hypertension Registry, SPAHR; European Society of Cardiology/European Respiratory Society, ESC/ERS). We compared the performance of artificial intelligence (AI) algorithms with conventional (non-AI) interpretation methods; the primary outcome was the area under the receiver operating characteristic curve (AUC), and secondary outcomes included sensitivity, specificity, summary receiver operating characteristic (SROC), and prevalence as reported.

### Exclusion criteria

We excluded studies for the following reasons: non-human research; case reports; case series; cross-sectional designs; editorials, reviews, comments; and conference abstracts without full text. We also excluded studies with no full text or with missing data that precluded outcome extraction, studies not involving PH populations or not performing risk stratification using the specified clinical, biomarker, imaging, hemodynamic, or risk-score domains, and studies that did not report AI-versus-conventional performance or failed to provide AUC, sensitivity, specificity, SROC, or prevalence required for synthesis.

### Data extraction

Two independent reviewers, HA and KB, performed data extraction using Microsoft Excel (version 16.0), and any discrepancies were resolved by a third, neutral reviewer, FH. The extracted data included all the key categories from the studies. These included author name, country, year of study, study design, AI algorithms used along with their parameters, and the traditional method of interpretation used. Patient characteristics that were recorded at baseline were mean age, sex, Right Heart Catheterization parameters (mean PAP, PVR, cardiac index, mean RAP, sPAP), and comorbidities. For the studies presenting binary classification results, we extracted the data in the form of a confusion matrix containing true positives, false positives, true negatives, and false negatives to analyze the pooled data.

### Quality assessment

The quality assessment was conducted by the QUADAS-2 tool. It has four domains, which are patient selection, index test, reference standard, and flow/timing, for the assessment of bias and concerns regarding applicability (Whiting et al., 2011). Each domain was classified as “low risk,” “some concerns,” or “high risk” based on the predetermined assessment criteria. Two independent reviewers did the assessments. The traffic light plot and summary plot are visible in (Supplementary Figures S1, S2).

### Statistical analysis

All statistical analyses were performed using R (version 2024.12.1 + 563) using 2 × 2 contingency data (Shim et al., 2019).

*Bivariate DTA Meta-Analysis:* A bivariate diagnostic random-effects meta-analysis was conducted using the Reitsma model via the reitsma function from the mada package to estimate the diagnostic summary and the summary receiver operating characteristic (SROC) curve. Due to package limitations (the mada package does not support bivariate forest plots), univariate analyses were performed separately.

*Univariate DTA Meta-Analysis:* The analysis was performed using the meta and metaprop functions in R (version 2024.12.1 + 563) using a logit transformation (PLOGIT) to stabilize variances (Shim et al., 2019). The Clopper–Pearson (CP) method was used to calculate confidence intervals, thereby ensuring accurate estimates of proportions. Forest plots for sensitivity, specificity, and DOR were generated using the forest function.

*Heterogeneity and Sensitivity Analysis:* To assess study heterogeneity, sensitivity analysis was performed using the metainf() function from the meta package.

*Publication Bias:* Potential publication bias was evaluated using funnel plots (funnel function, meta package). Egger’s test (metabias function, meta package).

*AUC:* Logit AUC conversion was performed before pooling using the metagen function in R. The general inverse variance method, combined with a restricted maximum likelihood (REML) model, was used to estimate between-study heterogeneity (I^2^). Sensitivity analyses, funnel plots, and Egger’s test were used to assess robustness and publication bias. Subgroup analyses were conducted based on the studies, which provided standard errors (SEs) and which had the imputed SEs because they were originally absent from the studies.

## Results

### Search results

**Figure 1:**
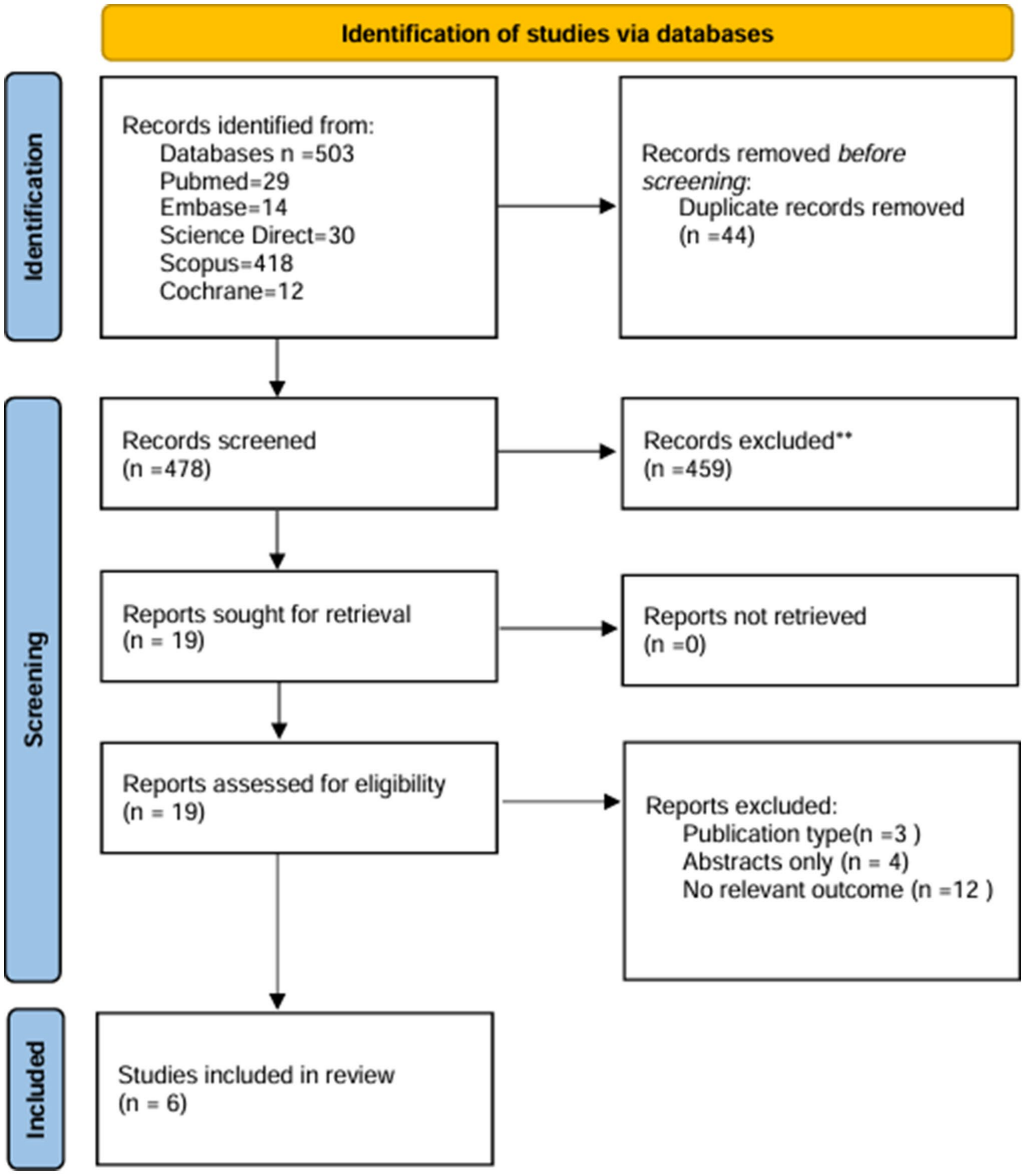
PRISMA flowchart.

We retrieved approximately 503 studies from databases such as PubMed, Science Direct, Cochrane, Scopus, and Embase. After removing 44 duplicates, 459 studies in Title/Abstract screening, and 13 studies in full text screening, we included 6 studies that matched our inclusion criteria. The details of the search results are mentioned in Figure 1.

### Study characteristics

All 6 studies included in this analysis had a cumulative sample size of 14,095 patients, with 4,481 patients in internal test data and 4,948 patients in external data. Additionally, the traditional method group had 1900 patients collectively. The age of patients ranged from 11 months to 74 years. The Random Forest Model was the most common AI algorithm model used, whereas REVEAL 2.0 is the most common traditional stratification tool used in these studies. Approximately 4,170 patients had idiopathic pulmonary hypertension, and 2,578 patients had some sort of connective tissue disease at baseline. The comprehensive data on the baseline of patients are mentioned in Table 1.

**Table 1:** Baseline characteristics.

Study ID	Country	Study design	AI- algorithms models	Traditional methods	Total No. of patients	AI-algorithms group—(n)		Traditional methods group—(n)	Mean age (years)	Male—n (%)	
						Internal test data	External test data			AI-algorithms group	Traditional group
Kanwar et al. (2020)	USA, Germany, Australia, Poland, Thailand	Retrospective Cohort Study	-PHORA –Bayesian Network (Tree-Augmented Naïve Bayes)	REVEAL 2.0	7,356	2,529	4,827	2529*	53.6	2096(28.4)	505 (20.0)
Kheyfets et al. (2023)	United States	Retrospective Cohort Study	-Random Forest Model	REVEAL 2.0	205	34	38	NA	49.7	44 (26.3)	NA
Duan et al. (2022)	China	Retrospective Cohort Study	AI models: -CatBoost -XGBoost -LightGBM -Random Forest	Traditional logistic regression	5,913	4,139	NA	1,774	0.11 (0.00, 0.60)	3,255 (55.04)	NA
Sonnweber et al. (2023)	Austria	Retrospective Cohort Study	-Elastic Net Cox regression -Partitioning Around Medoids (PAM)	- (FPHR) 3-parameter - (FPHR) 4-parameter (FPHR 4p) - (COMPERA) Score: - (SPAHR) Model - (mRASP)	183	100	83	NA	66 (IQR: 53–71) ** 70 (IQR: 54–74) ***	64 (35.09)	NA
Yang et al. (2023)	China	AI model development and validation (imaging-based) study	-Prior Prompt Network (P2-Net)	- Cox Proportional Hazards Model - Time-dependent ROC for classification Parameters Used: - Hazard ratios (HRs) - (C-index) - Log-rank test	140	NA	NA	36	NA	NA	NA
Ostermann et al. (2023)	Germany	Retrospective Cohort Study	-Least Absolute Shrinkage -Selection Operator (LASSO) Cox Regression Model	NA	298	208	NA	90	64 years (IQR 48–74)	115 (39)	NA

*REVEAL 2.0 and Internal Validation were used on the same database called the REVEAL 2.0 Registry. **These patients are combined WHO Performance Status I/II combined. ***These patients are combined WHO Performance Status III/IV combined. ****Internal Validation data. *****External Validation data. AI, artificial intelligence; n, number of patients; NA, not available or not applicable; PAP, Pulmonary Arterial Pressure; PVR, pulmonary vascular resistance; PAH, pulmonary arterial hypertension; IQR, Interquartile Range; mmHg, millimeters of mercury; and Wood unit, unit for PVR measurement. Scoring systems and registries include REVEAL 2.0 (Registry to Evaluate Early and Long-term PAH Disease Management); FPHR, French Pulmonary Hypertension Registry; COMPERA, Comparative; Prospective Registry of Newly Initiated Therapies for Pulmonary Hypertension; SPAHR, Swedish PAH Register; mRASP, Modified Risk Assessment Score of PAH; and PHORA, Pulmonary Hypertension Outcomes Risk Assessment. Machine learning models and techniques include Random Forest; CatBoost, XGBoost, LightGBM, Elastic Net Cox regression; LASSO, Least Absolute Shrinkage and Selection Operator; Bayesian Network (including Tree-Augmented Naïve Bayes); and P2-Net, Prior Prompt Network. Additional methods include PAM, partitioning around medoids; ROC, receiver operating characteristic; C-index, Concordance Index, and log-rank test used in survival analysis.

### Quality assessment

Quality Assessment was performed using QUADAS-2, which showcased the risk of bias and applicability concerns among all the included studies. Five out of six studies exhibited a low risk of bias (ROB) in all domains. However, Yang et al. (2023) highlighted a high risk of bias due to suboptimal performance in Domain 4 (flow and timing) of ROB. All the included studies (Kanwar et al., 2020; Kheyfets et al., 2023; Duan et al., 2022; Sonnweber et al., 2023; Yang et al., 2023; Ostermann et al., 2023) showed low applicability concerns as shown in Supplementary Figures S1, S2.

### Diagnostic test accuracy results

#### Area under curve

**Figure 2:**
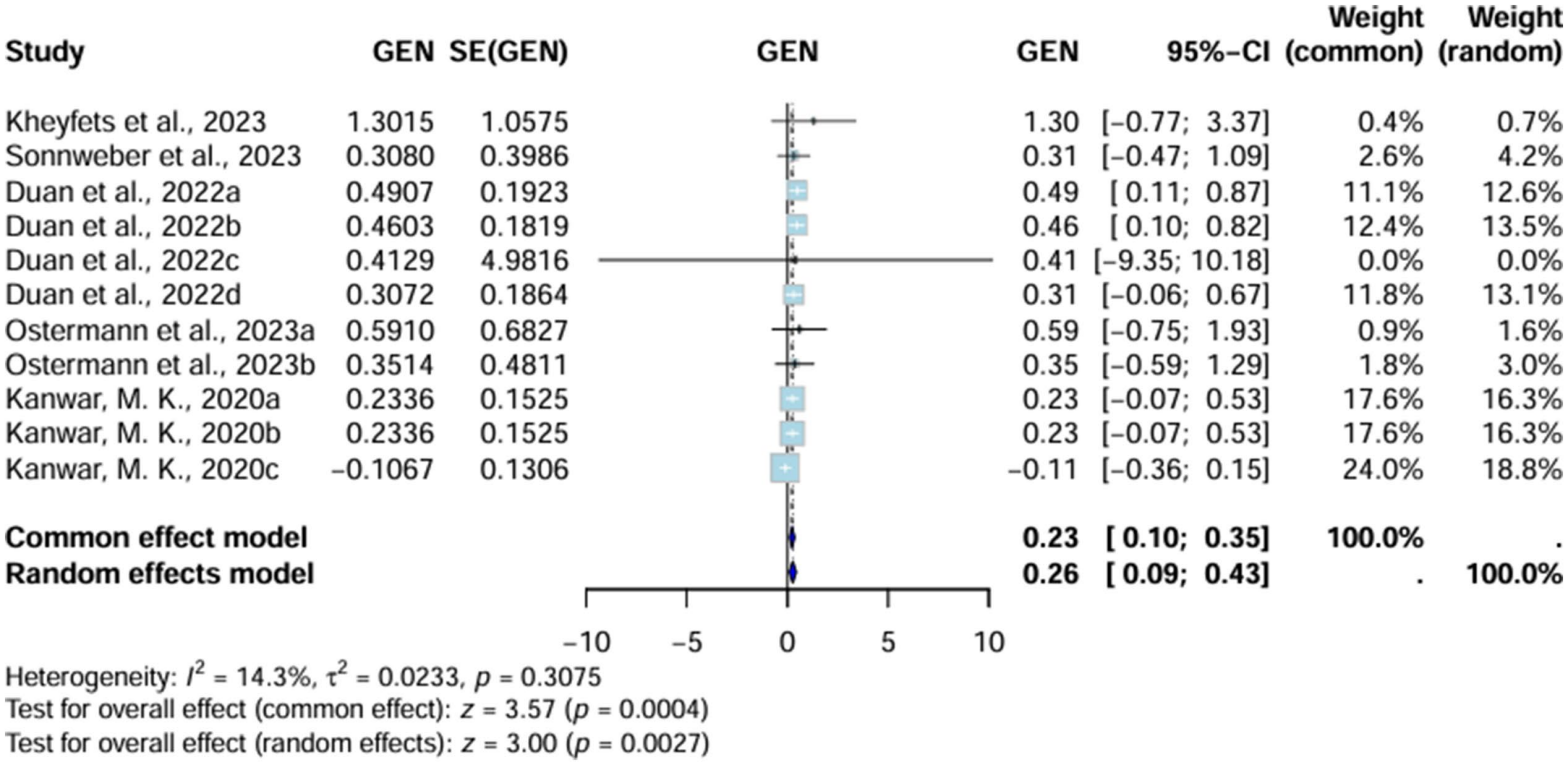
Forest plot shows pooled AUC across all studies.

Area under curve (AUC) significantly favors AI-algorithm models, with the random effects model reporting a value of 0.26 (95% CI 0.09–0.43) and the common effect model indicating up to 0.23 (95% CI 0.10–0.35). Moreover, heterogeneity (I^2^) is also low (14.3%, T^2^ = 0.0233, *p* = 0.3075), underscoring the consistency of these results across all studies included. However, the value of heterogeneity is non-significant (Figure 2).

##### Sensitivity analysis of AUC

Heterogeneity (I^2^) further decreases substantially with the omission of Duan et al. (2022), showing the role of these two studies in increasing the heterogeneity (I^2^) of our analysis (Supplementary Figure S3).

##### Subgroup analysis of AUC

All the studies were divided into two subgroups. The first included studies that reported their standard errors (SEs), while the second group comprised those for which SEs had to be imputed. Heterogeneity (I^2^) decreased to 0% (T^2^ = 0, *p* = 0.9853) in studies where SEs were provided. Studies in the imputed SE group showed a heterogeneity (I^2^) of 50.5% (T^2^ = 0.0214, *p* = 0.1327). This indicates that studies with imputed SEs contributed to increased heterogeneity. However, the heterogeneity (I^2^) values remained non-significant (Supplementary Figure S4).

#### Univariate analysis

##### Sensitivity

**Figure 3:**
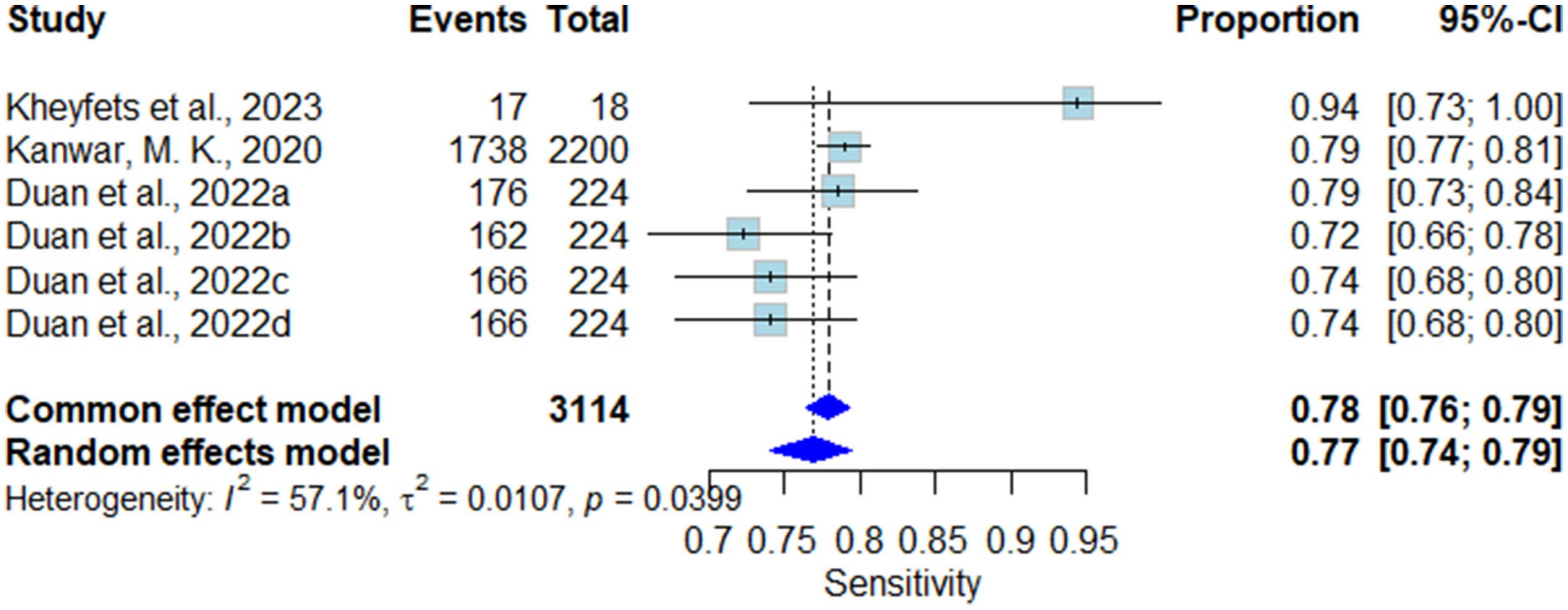
Forest plot for pooled sensitivity across all studies.

The random effect model showed a statistically significant pooled sensitivity of 0.77 (95% CI 0.74–0.79) in favor of AI-Algorithm Models across the included studies. These results were slightly different for the common effect model, estimating up to 0.78 (95% CI 0.75–0.79). The heterogeneity (I^2^) was modestly high in these results, accounting for up to 57.1% (T^2^ = 0.0107, *p* = 0.0399) (Figure 3).

##### Sensitivity analysis of pooled sensitivity

The sensitivity analysis on pooled sensitivity showed that by omitting (Kanwar et al., 2020) heterogeneity (I^2^) came down to 25.9%, therefore indicating it is the study responsible for increasing heterogeneity (I^2^). Moreover (Duan et al., 2022), omission causes the heterogeneity (I^2^) to go up to 65.4% therefore showing its effect on moderating the heterogeneity (Supplementary Figure S5).

##### Specificity

**Figure 4:**
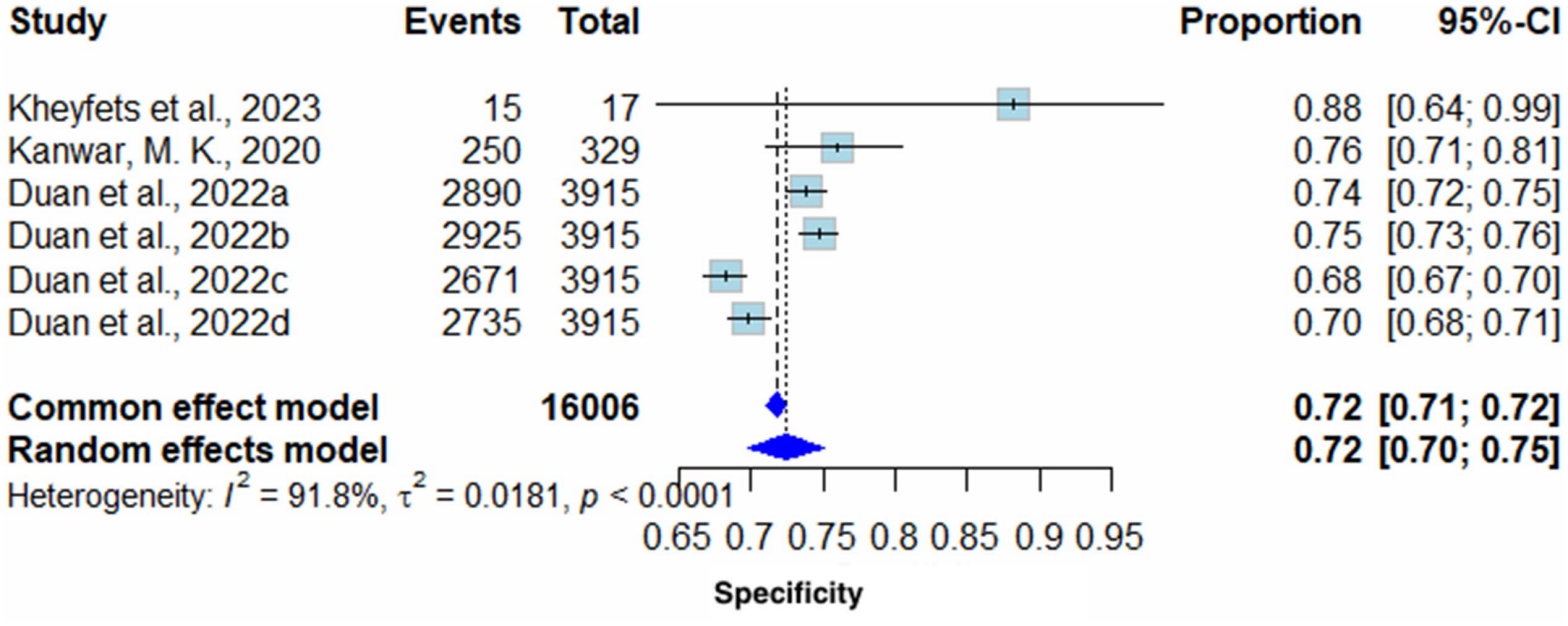
Forest plot for pooled specificity across all studies.

Pooled Specificity yielded on the random effects model was 0.72 (95% CI 0.70–0.75) in favor of AI algorithm models. The common effect model showed the same results with slightly different confidence intervals (0.72 (95% CI 0.71–0.72). Heterogeneity (I^2^) of these results was 91.8% (T^2^ = 0.0181, *p* < 0.0001), therefore, highlighting high variability across included studies (Figure 4).

##### Sensitivity analysis of pooled specificity

Excluding all sensitivity analysis studies had minimal effect on heterogeneity (I^2^); however, the removal of Duan et al. (2022) notably decreased heterogeneity to 86.6% (Supplementary Figure S6).

##### Diagnostic odds ratio

**Figure 5:**
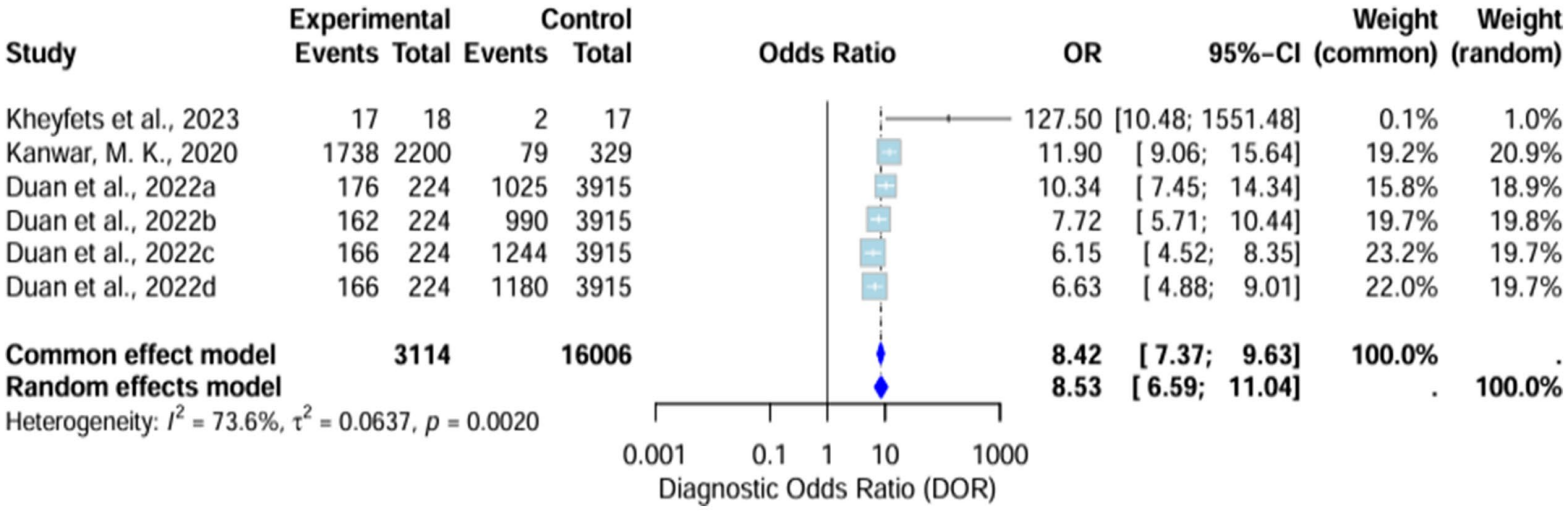
Forest plot for pooled diagnostic odds ratio (DOR) across all studies.

The diagnostic odds ratio (DOR) yielded was substantially high and significant in favor of AI algorithm models. The random effects model showed it around 8.53 (6.59–11.04), whereas the common effect model demonstrated it around 8.42 (7.37–9.63). The heterogeneity (I^2^) of these results was 73.6% (T^2^ = 0.0637, *p* = 0.0020), signifying high variability across the included studies (Figure 5).

##### Sensitivity analysis of pooled diagnostic odds ratio

Sensitivity analysis demonstrates that the omission of Kanwar et al. (2020) drops the heterogeneity (I^2^) to 63.3%, which remains high (Supplementary Figure S7).

#### Interpretation of receiver operating curve (ROC) plane plot and ROC curve

**Figure 6:**
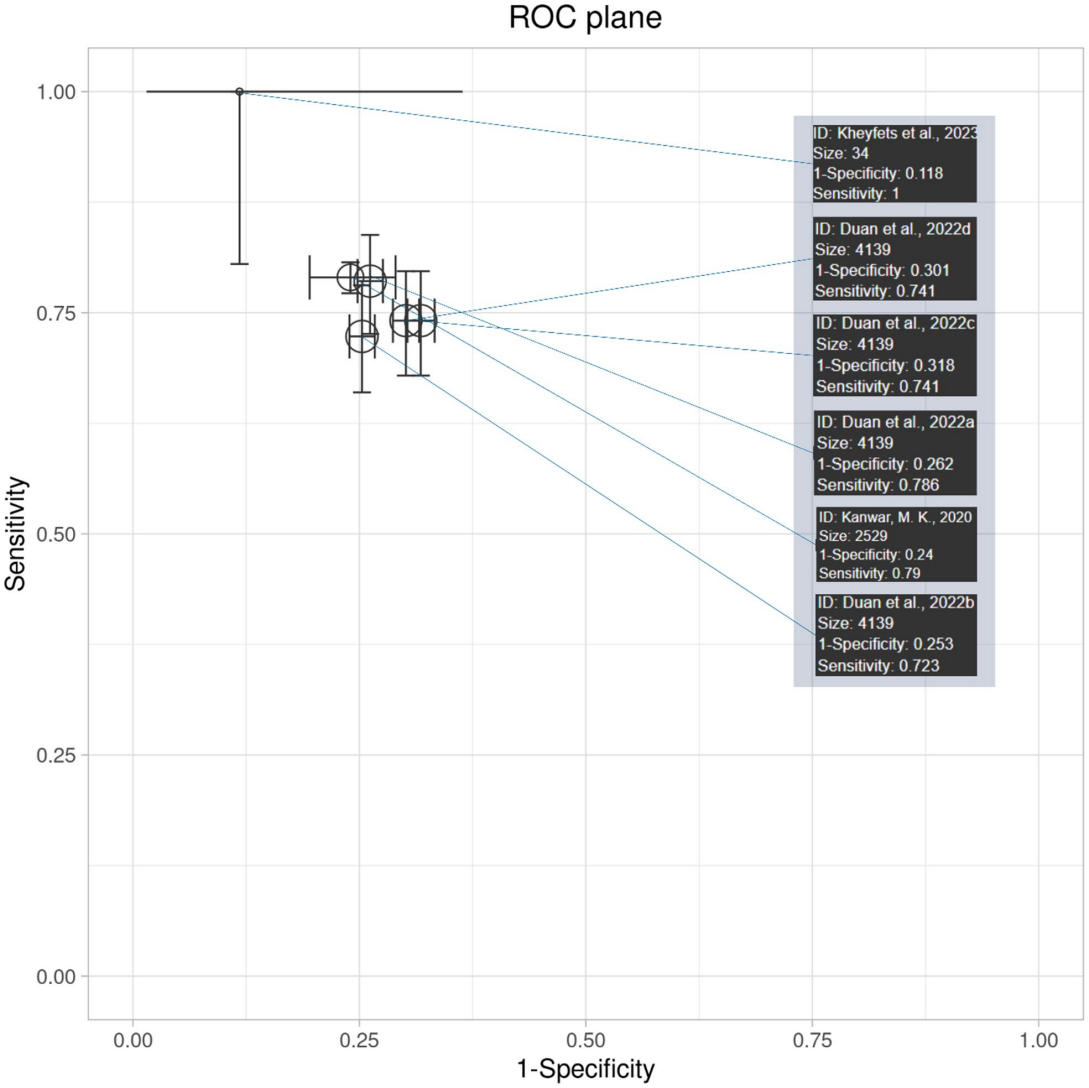
SROC plane with 95% confidence interval plot of all studies.

The ROC plane plot in Figure 6 shows the diagnostic accuracy and 95% confidence intervals of AI algorithm models across various studies. The majority of the studies lie in zones of moderate diagnostic accuracy, with sensitivity and specificity ranging from 70 to 80%. (Kheyfets et al., 2023) come out as an outlier with near-perfect sensitivity; however, its higher confidence interval highlights the limitation of its results. Overall, the plot demonstrates moderately high diagnostic accuracy, consistent across studies but with some variability in precision.

## Discussion

This meta-analysis aimed to assess the prognostic ability of AI algorithms in the prediction of the severity of disease, risk stratification by analyzing various parameters and risk scores among patients affected by PH. We evaluated the efficacy of AI models compared to conventional prognostic methods. The clinical effectiveness was assessed through different diagnostic performance metrics, including area under the curve (AUC), sensitivity, and specificity. The results of this study present both the advantages and challenges associated with implementing these methods into clinical practice.

The AI models were developed by analyzing patients’ data and the key information from various variables used in the risk stratification of PH patients (Vraka et al., 2023). In routine clinical practice, physicians use parameters such as 6MWD, CPET, Nt-pro BNP levels, and different echocardiography, RHC, and cardiac MRI variables to assess the severity and stage of the disease. In our study, the most commonly used variables in AI-driven PH risk stratification were 6MWD (Duan et al., 2022; Kheyfets et al., 2023; Sonnweber et al., 2023), NT-proBNP (Kheyfets et al., 2023; Sonnweber et al., 2023), PVR (Kanwar et al., 2020; Sonnweber et al., 2023), cardiac function markers, (cardiac index, Kanwar et al., 2020; Sonnweber et al., 2023), right atrial area (Kanwar et al., 2020; Sonnweber et al., 2023), and demographic factors age, (Duan et al., 2022; Sonnweber et al., 2023), gender (Duan et al., 2022; Kanwar et al., 2020), WHO functional class (Kanwar et al., 2020; Sonnweber et al., 2023) and the most commonly used risk scores were REVEAL 2.0 (Kanwar et al., 2020; Kheyfets et al., 2023), FPHR, SPAHR, and mRASP (Sonnweber et al., 2023) among AI-driven models and conventional prognosis methods.

### Strengths of AI models

Included AI-based stratification algorithms yielded promising diagnostic results across various prognostic metrics. The sensitivity of the AI models was 0.77, which is considered a good diagnostic performance. The application of ML methods could provide a faster diagnosis. Theoretically, AI models could predict the incidence of adverse events significantly faster than the use of standard prognostic tools. This could impact the treatment workflow, as early intervention for PH could significantly improve a patient’s quality of life. Pooled DOR yielded a result of 8.53, confirming its effectiveness in correctly identifying patients with the condition. The high diagnostic results were generally consistent across the included studies.

Moreover, the clinical value of AI algorithms yielded a statistically higher AUC compared to traditional risk scores, which confirmed the high prognostic accuracy of the computer-aided algorithm for PH. AUC remains a premier diagnostic outcome, as the higher the AUC, the more effective the algorithm is at the prognostic task.

### Variability and heterogeneity in results

However, although these strong prognostic findings are promising, there were notable differences among the studies included in this meta-analysis. High heterogeneity was observed in sensitivity (57.1%), specificity (91.8%), and diagnostic odds ratio (DOR) (73.6%). The inconsistency in these primary outcomes was significant, and several factors could impact this variability across the included studies. First, variations in the AI model itself could significantly impact the final diagnostic performance. Differences in terms of training and validation set sizes, along with validation methods (traditional split, k-cross validation, or external testing of the model), could determine whether the model was trained sufficiently to provide a reliable diagnosis (Sivakumar et al., 2024). Moreover, the algorithm structure itself could be dependent on parameter tuning, optimization algorithms, or the learning rate and technique of the model. The selection of variables for model prediction was another aspect that determined prognostic performance. Studies could adjust different priorities for given clinical variables, leading to differences across the models. Finally, variations in patient demographics could theoretically lead to different prevalence of adverse events across the studies, making diagnosis more challenging across subpopulations where adverse events occurred less.

### Challenges

Currently, no direct head-to-head meta-analysis exists comparing AI-based and traditional risk stratification methods for patients with PH, primarily due to the limited number of available studies. Existing research utilizes various models developed with different clinical variables, and physicians worldwide use diverse risk stratification approaches, resulting in considerable heterogeneity. However, in recent studies, AI models demonstrated greater diagnostic performance for the diagnosis of PH, especially those analyzing cardiac MRI (Hardacre et al., 2021), echocardiography (Salehi et al., 2025), and clinical biomarkers (Fadilah et al., 2024), compared with conventional methods.

Finally, ethical concerns remain a leading issue. The use of private patient data should be appropriately secured and must not be leaked outside hospital facilities. This might be especially challenging in multi-institutional models, where data are transferred through the Internet, and there is a risk of sensitive data leaks. There are also important barriers to translating AI-assisted technology into clinical medicine, including integration into clinical workflows, physician acceptance, and regulatory approval (Nair et al., 2024). Studies show that even accurate AI tools may be met with resistance due to liability concerns, the need for robust evidence, and ethical considerations in patient care (Ahmed et al., 2023).

## Limitations

This study has several limitations. First, the high inconsistency observed among AI models may be due to differences in the number of validation sets, the clinical variables used, and differences in algorithms, all of which limit the generalizability of the findings. Although these models demonstrate high diagnostic performance, such variability raises important concerns about their reliable adoption in clinical practice. Second, the heterogeneous PH patient population also contributed to the high heterogeneity. Third, only six studies were included due to limited literature directly addressing the prognostic accuracy of AI-based risk stratification and traditional methods in PH patients. This small sample size may produce an underpowered analysis, emphasizing the need for more high-quality, large-scale studies for robust comparisons. Finally, limited external validation and the “black box” nature of AI models present challenges for objectively assessing performance and hinder clinical reliability, underscoring the need for more transparent, explainable approaches in future studies.

## Implications for future research

The results of this study open the door to future paths, which may lead to the integration of AI algorithms into routine clinical practice. Naturally, more studies concerning PH management are needed to fully validate the prognostic ability, especially across diverse clinical settings. The use of convolutional neural networks and deep learning-based families of algorithms could provide even more accurate results compared to traditional ML models. However, such investments typically require significantly larger training and validation set sizes to accurately train the model for such tasks. The inclusion of various clinical variables, multimodal imaging sources, and multiple AI algorithms could improve the management of PH. Standardization of methodologies is needed to provide more robust comparisons of the diagnostic performance. Economic analysis could potentially analyze how these AI-based algorithms impact the management of PH and whether there can be any cost savings with the use of computer-based prognosis. Finally, higher-quality trials are needed, as currently included studies possess bias concerns. Future trials should consider key variables such as 6MWD, NT-proBNP, PVR, cardiac function markers (e.g., cardiac index and right atrial area), and demographic factors (age, gender, and the WHO functional class), along with validated risk scores such as REVEAL 2.0, FPHR, SPAHR, and mRASP, to enhance comparability and clinical relevance.

## Conclusion

AI algorithm models showed superiority in risk stratification, prognostication, and severity assessment of the PH patients. The AUC was significantly higher for AI models compared to traditional methods, with the results showing limited variability across all included studies. Sensitivity, specificity, and diagnostic odds ratio were also higher than those of traditional methods. However, challenges such as limited external validation, inconsistent results, and ethical concerns must be addressed. Future research should focus on robust, transparent, and secure AI applications to ensure clinical reliability.

## Data Availability

The original contributions presented in the study are included in the article/Supplementary material, further inquiries can be directed to the corresponding author.

## References

[ref1] Ahmed A. Ahmed S. Rådegran G. (2023). Risk assessment in pulmonary arterial hypertension: a step towards clinical implementation based on the 2022 ESC/ERS pulmonary hypertension guidelines. Pulm Circ. 13:e12253. doi: 10.1002/pul2.12253, PMID: 37332852 PMC10271592

[ref2] Ahmed M. I. Spooner B. Isherwood J. Lane M. Orrock E. Dennison A. (2023). A systematic review of the barriers to the implementation of artificial intelligence in healthcare. Cureus. 15:e46454. doi: 10.7759/cureus.46454, PMID: 37927664 PMC10623210

[ref3] Attaripour Esfahani S. (2025). A comprehensive review of artificial intelligence (AI) applications in pulmonary hypertension (PH). Medicina 61:85. doi: 10.3390/medicina61010085, PMID: 39859065 PMC11766811

[ref4] Benza R. L. Kanwar M. K. Raina A. (2021). Development and validation of an abridged version of the REVEAL 2.0 risk score calculator, REVEAL lite 2, for use in patients with pulmonary arterial hypertension. Chest 159, 337–346. doi: 10.1016/j.chest.2020.08.2069, PMID: 32882243 PMC7462639

[ref5] Duan M. Shu T. Zhao B. Xiang T. Wang J. Huang H. . (2022). Explainable machine learning models for predicting 30-day readmission in pediatric pulmonary hypertension: a multicenter, retrospective study. Front. Cardiovasc Med. 9:919224. doi: 10.3389/fcvm.2022.919224, PMID: 35958416 PMC9360407

[ref6] Fadilah A. Putri V. Y. S. Puling I. M. D. R. Willyanto S. E. (2024). Assessing the precision of machine learning for diagnosing pulmonary arterial hypertension: a systematic review and meta-analysis of diagnostic accuracy studies. Front. Cardiovasc Med. 11:1422327. doi: 10.3389/fcvm.2024.1422327, PMID: 39257851 PMC11385608

[ref7] Hardacre C. J. Robertshaw J. A. Barratt S. L. Adams H. L. MacKenzie Ross R. V. Robinson G. R. . (2021). Diagnostic test accuracy of artificial intelligence analysis of cross-sectional imaging in pulmonary hypertension: a systematic literature review. Br. J. Radiol. 94:20210332. doi: 10.1259/bjr.20210332, PMID: 34541861 PMC8631018

[ref9] Imai S. (2024). Artificial intelligence-based model for predicting pulmonary arterial hypertension on chest x-ray images. BMC Pulm. Med. 24:101. doi: 10.1186/s12890-024-02891-4, PMID: 38413932 PMC10898025

[ref10] Kanwar M. K. Gomberg-Maitland M. Hoeper M. Pausch C. Pittrow D. Strange G. . (2020). Risk stratification in pulmonary arterial hypertension using Bayesian analysis. Eur. Respir. J. 56:2000008. doi: 10.1183/13993003.00008-2020, PMID: 32366491 PMC7495922

[ref11] Kheyfets V. O. Sweatt A. J. Gomberg-Maitland M. Ivy D. D. Condliffe R. Kiely D. G. . (2023). Computational platform for doctor-artificial intelligence cooperation in pulmonary arterial hypertension prognostication: a pilot study. ERJ Open Res. 9, 00484–02022. doi: 10.1183/23120541.00484-2022, PMID: 36776484 PMC9907150

[ref12] McInnes M. D. F. Moher D. Thombs B. D. McGrath T. A. Bossuyt P. M. Group P-D (2018). Preferred reporting items for a systematic review and meta-analysis of diagnostic test accuracy studies: the PRISMA-DTA statement. JAMA 319, 388–396. doi: 10.1001/jama.2017.19163, PMID: 29362800

[ref13] McLaughlin V. V. (2024). Riociguat improves long-term outcomes and COMPERA 2.0 risk status in PAH. Respir. Med. 215:107910.

[ref14] Nair M. Svedberg P. Larsson I. Nygren J. M. (2024). A comprehensive overview of barriers and strategies for AI implementation in healthcare: mixed-method design. PLoS One 19:e0305949. doi: 10.1371/journal.pone.0305949, PMID: 39121051 PMC11315296

[ref15] Oliveros E. Jonnalagadda A. Vaidya A. (2025). Pulmonary hypertension: updates in diagnosis and management. J. Clin. Med. 14:2400. doi: 10.3390/jcm14072400, PMID: 40217850 PMC11989491

[ref16] Ostermann J. Pott J. Hennigs J. K. Roedl K. Sinning C. Harbaum L. . (2023). Residual risk identified in routine noninvasive follow-up assessments in pulmonary arterial hypertension. ERJ Open Res. 9, 00072–02023. doi: 10.1183/23120541.00072-2023, PMID: 37260464 PMC10227628

[ref17] Park S. (2023). CT-based AI model for lung fibrosis quantification predicts mortality in PAH. Radiology.

[ref8] Pausch C. Pittrow D. Hoeper M. M. Huscher D. (2023). Performance of the ESC/ERS 4-strata risk stratification model for pulmonary arterial hypertension with missing variables. The European respiratory journal, 62:2301023. doi: 10.1183/13993003.01023-2023 37802633 PMC10695769

[ref18] Salehi M. Alabed S. Sharkey M. Maiter A. Dwivedi K. Yardibi T. . (2025). Artificial intelligence-based echocardiography assessment to detect pulmonary hypertension. ERJ Open Res. 11, 00592–02024. doi: 10.1183/23120541.00592-2024, PMID: 40356796 PMC12067425

[ref19] Shim S. R. Kim S. J. Lee J. (2019). Diagnostic test accuracy: application and practice using R software. Epidemiol Health. 41:e2019007. doi: 10.4178/epih.e2019007, PMID: 30999739 PMC6545496

[ref20] Siontis K. C. (2022). Deep learning electrocardiogram model for detection of elevated pulmonary artery pressure. J Am Coll Cardiol AI. 1, 223–234.

[ref21] Sivakumar M. Parthasarathy S. Padmapriya T. (2024). Trade-off between training and testing ratio in machine learning for medical image processing. PeerJ Comput. Sci. 10:e2245. doi: 10.7717/peerj-cs.2245, PMID: 39314694 PMC11419616

[ref22] Sonnweber T. Tymoszuk P. Steringer-Mascherbauer R. Sigmund E. Porod-Schneiderbauer S. Kohlbacher L. . (2023). The combination of supervised and unsupervised learning based risk stratification and phenotyping in pulmonary arterial hypertension—a long-term retrospective multicenter trial. BMC Pulm. Med. 23:143. doi: 10.1186/s12890-023-02427-2, PMID: 37098543 PMC10131314

[ref23] Vraka A. Diamanti E. Kularatne M. Yerly P. Lador F. Aubert J. D. . (2023). Risk stratification in pulmonary arterial hypertension, update and perspectives. J. Clin. Med. 12:4349. doi: 10.3390/jcm12134349, PMID: 37445381 PMC10342910

[ref24] Whiting P. F. Rutjes A. W. S. Westwood M. E. Mallett S. Deeks J. J. Reitsma J. B. . (2011). QUADAS-2: a revised tool for the quality assessment of diagnostic accuracy studies. Ann. Intern. Med. 155, 529–536. doi: 10.7326/0003-4819-155-8-201110180-00009, PMID: 22007046

[ref25] Yang G. He Y. Lv Y. Chen Y. Coatrieux J. L. Sun X. . (2023). Multi-task learning for pulmonary arterial hypertension prognosis prediction via memory drift and prior prompt learning on 3D chest CT. IEEE J. Biomed. Health Inform. 27, 1967–1978. doi: 10.1109/JBHI.2023.3247492, PMID: 37027678

