# Supplementary material for: Comparative diagnostic accuracy of artificial intelligence-derived risk stratification versus conventional risk stratification methods in pulmonary hypertension patients: a systematic review and meta-analysis
Source: Front Artif Intell. 2025 Nov 19;8:1692829. doi: 10.3389/frai.2025.1692829 (PMC12673395; doi:10.3389/frai.2025.1692829)

**Table 1: Supplementary search**

| Pubmed | 29 | (("AI algorithm*"[tw] OR "artificial intelligenc*"[tw] OR "machine learning*"[tw] or "Deep Learning*"[tw] OR "Natural Language Processing*"[tw] OR "Machine Learning"[Mesh] OR "Artificial Intelligence"[Mesh] OR "Artificial Intelligence"[Mesh] OR "Natural Language Processing"[Mesh]) AND ("Pulmonary hypertension"[tw] OR "Pulmonary arterial hypertension"[tw] OR "Primary pulmonary hypertension"[tw] OR "Secondary pulmonary hypertension"[tw] OR "Pulmonary vascular disease*"[tw] OR "Hypertension, Pulmonary"[Mesh] OR "Pulmonary Arterial Hypertension"[Mesh])) AND ("Risk Stratification*"[tw] OR "risk assessment*"[tw] OR "risk categorization*"[tw] OR "risk classification*"[tw] OR "risk grouping*"[tw] OR "risk evaluation*"[tw] OR "Risk analysis*"[tw] OR "Risk Assessment"[Mesh]) |
| --- | --- | --- |
| Embase | 14 | ('AI algorithm' OR 'artificial intelligence' OR 'machine learning' or 'Deep Learning' OR 'Natural Language Processing') AND ('Pulmonary hypertension' OR 'Pulmonary arterial hypertension' OR 'Primary pulmonary hypertension' OR 'Secondary pulmonary hypertension' OR 'Pulmonary vascular disease') AND ('Risk Stratification' OR 'risk assessment' OR 'risk categorization' OR 'risk classification' OR 'risk grouping' OR 'risk evaluation' OR 'Risk analysis') |
| Science direct | 30 | ('artificial intelligence' OR 'Deep Learning' OR 'machine learning' OR 'AI algorithm') AND ('Pulmonary hypertension' OR 'Pulmonary arterial hypertension') AND ('Risk Stratification' OR 'risk assessment' OR 'Risk analysis') |
| Scopus | 418 | ( &apos;artificial AND intelligence&apos; OR &apos;deep AND learning&apos; OR &apos;machine AND learning&apos; OR &apos;ai AND algorithm&apos; ) AND ( &apos;pulmonary AND hypertension&apos; OR &apos;pulmonary AND arterial AND hypertension&apos; ) AND ( &apos;risk AND stratification&apos; OR &apos;risk AND assessment&apos; OR &apos;risk AND analysis&apos; ) AND ( LIMIT-TO ( DOCTYPE , "ar" ) ) |
| Cochrane | 12 | ID Search Hits  #1 (AI algorithm):ti,ab,kw 323  #2 (artificial intelligence):ti,ab,kw 2578  #3 (machine learning):ti,ab,kw 3266  #4 (Deep Learning):ti,ab,kw 1708  #5 (Natural Language Processing):ti,ab,kw 323  #6 MeSH descriptor: [Artificial Intelligence] explode all trees 3388  #7 MeSH descriptor: [Machine Learning] explode all trees 1082  #8 MeSH descriptor: [Deep Learning] explode all trees 356  #9 MeSH descriptor: [Natural Language Processing] explode all trees 76  #10 #1 OR #2 OR #3 OR #4 OR #5 OR #6 OR #7 OR #8 OR #9 8477  #11 (Pulmonary hypertension):ti,ab,kw 6427  #12 (Pulmonary arterial hypertension):ti,ab,kw 2892  #13 (Pulmonary vascular disease):ti,ab,kw 2013  #14 MeSH descriptor: [Hypertension, Pulmonary] explode all trees 1682  #15 MeSH descriptor: [Pulmonary Arterial Hypertension] explode all trees 208  #16 #11 OR #12 OR #13 OR #14 OR #15 7566  #17 (Risk Stratification):ti,ab,kw 5597  #18 (risk assessment):ti,ab,kw 84278  #19 (risk evaluation):ti,ab,kw 34820  #20 (Risk analysis):ti,ab,kw 139106  #21 MeSH descriptor: [Risk Assessment] explode all trees 13456  #22 MeSH descriptor: [Risk Assessment] explode all trees 13456  #23 #17 OR #18 OR #19 OR #20 OR #21 OR #22 191855  #24 #10 AND #16 AND #23 12 |

**Figure 2- Risk of bias domains**


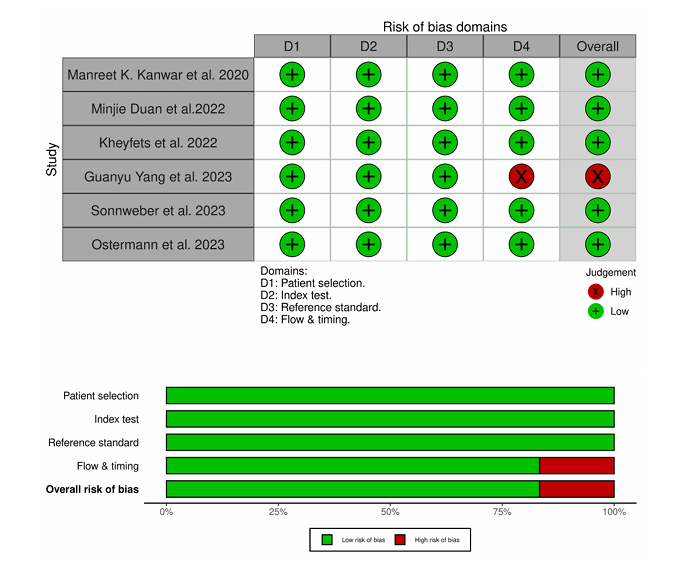


**Figure 3- Risk of bias and applicability concern assessed via QIADAS-2 tool**


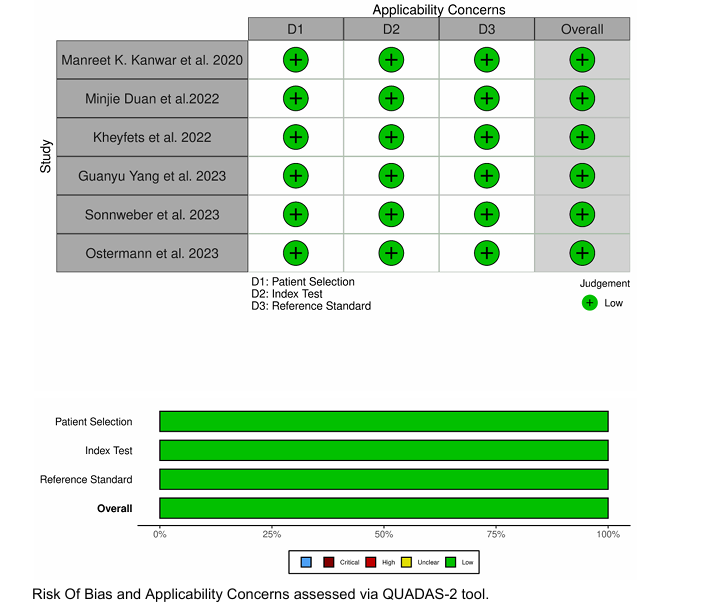


**Figure 3- Forest plot of sensitivity analysis of AUC**


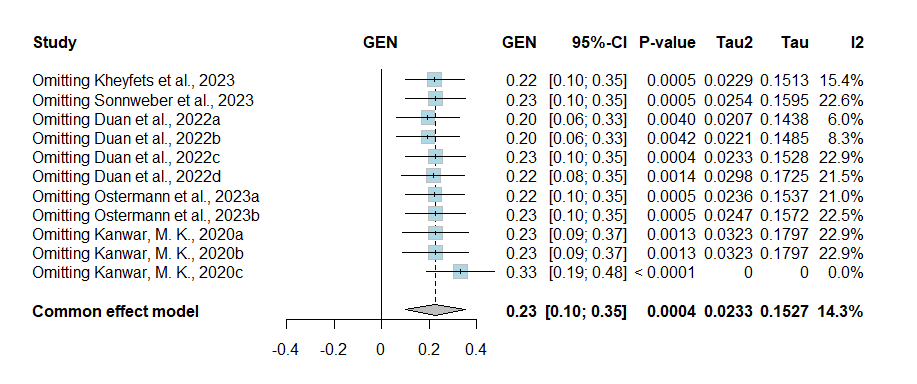


**Figure 4- Forest plot indicating subgroup analysis of AUC**


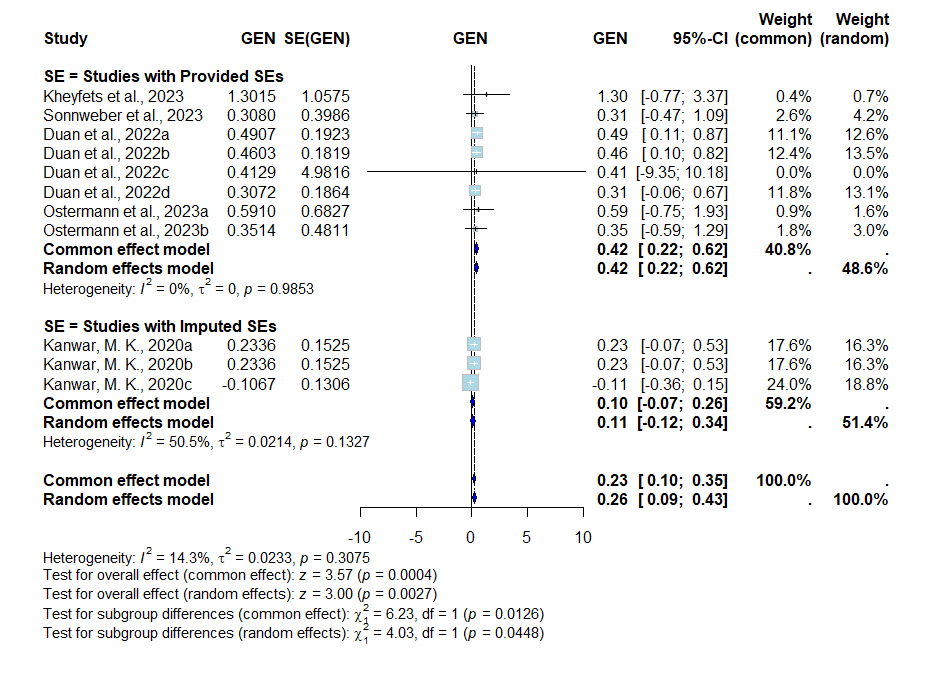


**Figure 5- Forest plot for sensitivity analysis on pooled sensitivity**


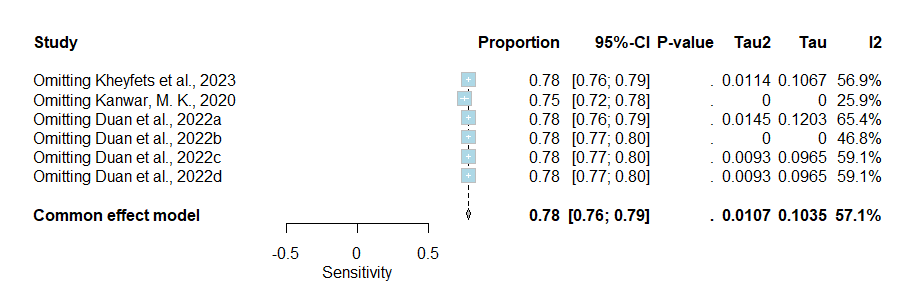


**Figure 6- Forest plot for Sensitivity analysis of pooled specificity**


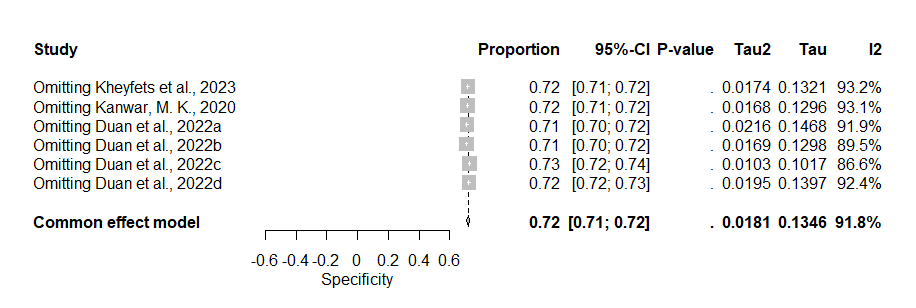


**Figure 7- Forest plot for sensitivity analysis of pooled Diagnostic Odds Ratio (DOR)**


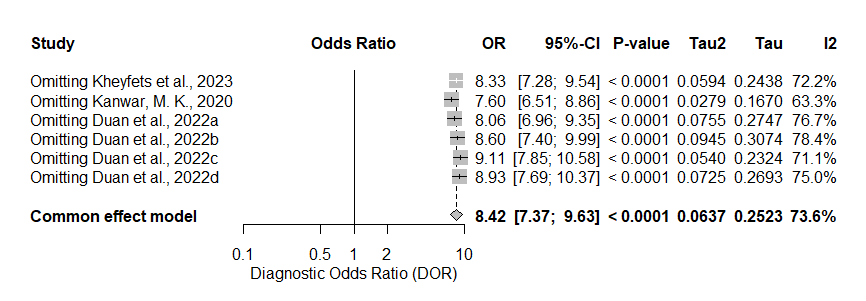

Supplement: Supplementary file 1 [file Data_Sheet_1.docx]
